# Streptococcus Pyogenes Epiglottitis in a Child: A Case Report

**DOI:** 10.7759/cureus.24123

**Published:** 2022-04-13

**Authors:** Massiel Apuy, Adriana Yock-Corrales, Ana Maria Moreno, Andrea Gutierrez

**Affiliations:** 1 Pediatrics, Hospital Nacional de Niños "Dr. Carlos Sáenz Herrera", San Jose, CRI; 2 Pediatric Emergency Medicine, Hospital Nacional de Niños "Dr. Carlos Sáenz Herrera", San Jose, CRI

**Keywords:** stridor, upper airway, respiratory distress, streptococcus pyogenes, epiglottitis

## Abstract

Acute epiglottitis in children is a rare entity since the introduction of the vaccine against *Haemophilus influenzae*; however, it should be considered as part of the differential diagnosis when facing a patient with evidence of upper airway obstruction. This study describes the case of a three-year-old child who arrived at the emergency department with fever, respiratory distress, and stridor. After ventilatory failure, the patient was intubated and antibiotics were initiated. The results of the bacteria culture confirmed *Streptococcus pyogenes *infection. This case report intends to describe and review the differential diagnoses of epiglottitis, as well as its management and prognosis.

## Introduction

Acute epiglottitis is the inflammation of the epiglottis and its adjacent structures. This entity can lead to abrupt blockage of the upper airway, acute ventilatory failure, and even death. *Haemophilus influenzae type b *(Hib) was the most frequent causative agent in pediatric patients before the implementation of the vaccine. Due to widespread vaccine compliance, the etiological agents include other types of *H. influenzae, Streptococci, Streptococcus aureus, *and *Streptococcus pyogenes*, among others [1,2].

Before the introduction of vaccination in the 1980s, there were an estimated 20,000 cases per year of invasive *H. influenzae *disease in the United States, with 17% related to epiglottitis [2]. After the implementation of the vaccine as part of the routine schedule, hospitalizations decreased by 91% between 1995 and 2003 [3]. Despite vaccination, epiglottitis in children has not disappeared because not all the population is vaccinated against *H. influenzae* and this disease can be caused by other infectious agents [4]. Even though the incidence of epiglottitis due to *H. influenzae* has decreased, this agent continues to be the most prevalent, followed by *S. pneumoniae, S. aureus,* and *S. pyogenes* [5].

## Case presentation

A three-year-old male known to be healthy arrived at the emergency department (ED) with a history of fever, vomit, and progressive shortness of breath. According to his mother, the patient did not have any suggestive event of choking or previous episodes of respiratory distress. The patient was febrile, with nasal flaring, suprasternal and intercostal retractions and low pitched inspiratory stridor. No sialorrhea or abnormal breath sounds were described on admission and he had normal heart and abdominal examinations. It was also noted that the patient presented a slight hyperextension of the neck and an oral breathing pattern.

During his admission to the ED, he presented recurrent non-bloody or bilious vomit and signs of mild dehydration. A bolus of normal saline solution was administered and, due to the possibility of severe croup, he was treated with a dose of IV dexamethasone at 0.6 mg/kg and nebulized adrenaline. The patient continued to have respiratory distress, so a high-flow nasal cannula (HFNC) was placed and administered along with nebulized budesonide, which resulted in an initial improvement of his respiratory pattern. The patient was stabilized and transferred to the intensive care unit.

**Figure 1:**
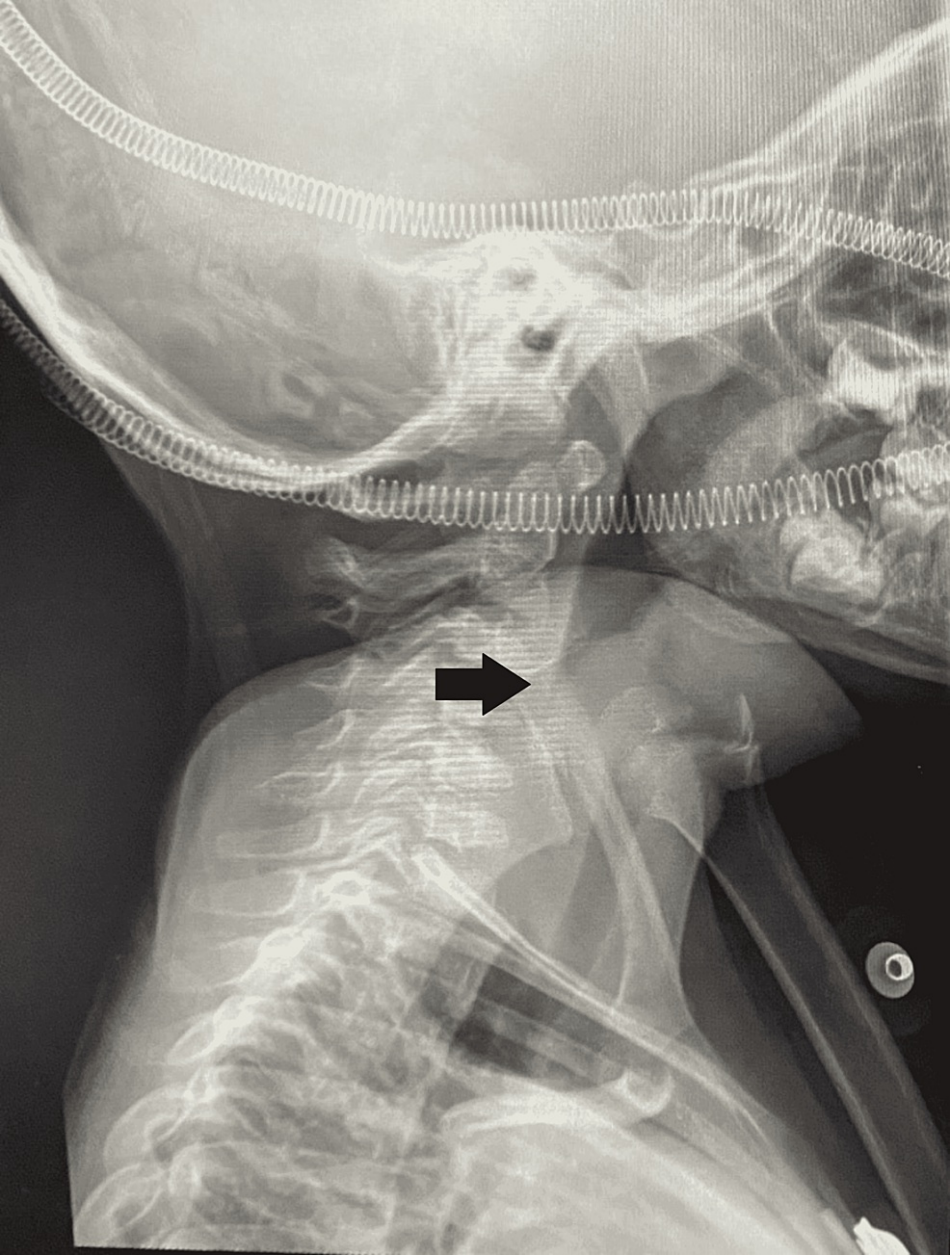
Lateral radiograph of the neck

Laboratory studies completed prior to his transfer included a complete blood count showing leukocytosis with predominantly polymorphonuclear and a slightly elevated c-reactive protein. A lateral neck x-ray was also performed and a "thumb sign" image suggestive of the enlarged epiglottis was identified (Figure 1).

In the ICU, the patient got worse and nasotracheal intubation was required. An edematous epiglottis with yellowish secretions was observed during the procedure. Blood cultures and bronchoalveolar lavage were taken, and empirical antibiotic coverage was initiated with cefotaxime and clindamycin. Subsequently, bronchoalveolar lavage culture was positive for *S. pyogenes, *which was sensitive to cefotaxime, so clindamycin was discontinued.

After 48 hours, the patient got better and was weaned off ventilatory support. He persisted with significant upper airway edema, so nasotracheal intubation was kept for supplemental oxygen for four days. Extubation was performed without complications, the patient was discharged after 10 days of IV cefotaxime with recommendations and follow-up with the pediatrician in his area.

## Discussion

Epiglottitis should still be considered in the differential diagnosis (infectious and non-infectious) of any child who presents with acute upper airway obstruction. Non-infectious causes include anatomical variations, foreign bodies, and trauma, among others. If the patient, in addition to the upper airway obstruction, presents with fever, elevation of acute phase reactants, or any other evidence of sepsis, an infectious bacterial agent should be considered as the primary infectious cause. In this case, diagnoses such as epiglottitis, laryngotracheobronchitis, abscesses, and tracheitis should be ruled out [2,6,7]. Table 1 shows a differential diagnosis of epiglottitis [7].

**Table 1:** Differential diagnosis of epiglottitis CBC: complete blood count; CT: computed tomography

Condition	Typical age range	Presentation	Diagnostic test
Epiglottitis	3 to 12 years	Acute onset of dysphagia, odynophagia, drooling, high fever, anxiety, and muffled voice	Neck radiography (thickened epiglottis) and CBC
Bacterial tracheitis	< 6 years	High fever, barking cough, respiratory distress, and rapid deterioration	Neck radiography (irregular tracheal mucosa) and CBC
Croup	6 months to 3 years	Acute onset of barking cough, stridor, and hoarseness	None required
Foreign body aspiration	< 3 years	Acute onset of choking and/or drooling	Neck radiography, neck CT, and airway endoscopy
Hemangioma	< 6 months	Stridor worse with crying	Airway endoscopy
Peritonsillar abscess	6 months to 3.5 years	Sore throat, fever, “hot potato” voice	Neck radiography, neck CT, and CBC
Retropharyngeal abscess	2 to 4 years	Fever, drooling, dysphagia, odynophagia, and neck pain	Neck radiography (bulging posterior pharyngeal wall), neck CT, and CBC
Thermal injury/smoke inhalation	No age predilection	Exposure to heat, smoke, or chemical	Direct laryngoscopy

*H. influenzae type b *(Hib) was the most frequent causative agent of epiglottitis in pediatric patients before implementation of vaccine [2]. Epiglottitis can still occur despite vaccination, and early recognition in the emergency department at arrival is lifesaving when treatment is given in a timely manner. Prior introduction of vaccine, epiglottitis primarily affected children between two and six years old. Incidence of epiglottitis is less frequent below the age of 18 months and more frequent in middle age and elderly patients [5].

Children with epiglottitis usually have a toxic appearance and present with fever, difficulty swallowing, sore throat, and neck moving restriction. As the infection progresses, a child may show behavior such as sitting upright, leaning forward, and being very still but breathing rapidly; drooling may occur because the child cannot swallow his or her own saliva and muffled or "hot potato" voice [6,8]. Altered mental status, mottled skin, and cyanosis are signs of airway obstruction and respiratory failure [5].

Imaging studies are not considered necessary for the diagnosis; however, there are some signs that may be useful. In presence of epiglottitis, the "thumb sign" can be found on the lateral neck radiograph, secondary to the thickening of the epiglottis and the aryepiglottic folds that have the appearance and approximate size of an adult thumb. On the other hand, the presence of upper airway edema triggers a loss of convexities at the subglottic level, which is seen as narrowing of the air column, known as the "hourglass sign" on an anteroposterior radiograph, and can be seen in croup as well as in epiglottitis, angioedema or bacterial tracheitis [2].

On arrival to the ED, the patient with possible epiglottitis should remain in a position of comfort. The first step in the approach is airway management, since the patient can deteriorate rapidly, and this includes minimal manipulation of the throat. Endotracheal intubation has proven to be one of the measures that reduce mortality compared to expectant management, especially in the pediatric population, when is performed early [2,9].

Securing the airway is critical. Intubation should be performed by the most experienced team member in order to manipulate the airway as little as possible and ideally in the operating room if possible. Supraglottic airway devices such as laryngeal mask should be avoided. In case intubation cannot be performed successfully, an emergency cricothyroidotomy should be performed. Fortunately, this is not a very frequent procedure [10]. Once airway is secure, cultures of the epiglottis, blood cultures, white blood cell count, electrolytes, and arterial blood gas should also be performed [5].

Antibiotic therapy is essential and should be given for seven to 14 days. A third-generation cephalosporin associated with an anti-staphylococcal agent is recommended as empirical therapy and always takes into account resistance rates and local prevalence [11]. The use of corticosteroids in these cases may be controversial, but they are still widely used. The patient should be transferred to an intensive care unit. The prognosis of these patients depends significantly on early diagnosis and clinicians should maintain a high index of suspicion when approaching a critically ill patient with upper airway obstruction [9,10].

## Conclusions

Epiglottitis is an inflammation of the epiglottis that can lead to airway obstruction, ventilatory failure, and death. It is a rare diagnosis in pediatric patients after the introduction of the Hib vaccine; however, there are still etiological agents that can cause this pathology. Despite this, a high index of suspicion should be maintained when a patient presents symptoms such as fever, respiratory distress, toxic appearance, and muffled voice, in order to reach an early diagnosis and optimal and immediate treatment, which will ultimately improve greatly the survival rate and long-term prognosis of the patient. In terms of treatment, it is of vital importance to secure the airway if necessary and antibiotic therapy remains essential. These patients should be managed if possible in an intensive care unit and upon discharge with follow-up by a specialist.
